# Molecular identification and DNA barcoding of *Chaenomeles japonica* in Pakistan

**DOI:** 10.1371/journal.pone.0348503

**Published:** 2026-05-05

**Authors:** Mohammad Islam, Sajjad Sajjad, Yehia Hazzazi, Mari Sumayli, Suad Alruzayza, Khushi Muhammad, Sajidul UI Ghafoor, Tufail Muhammad, Inamullah Ullah, A. El-Shabasy

**Affiliations:** 1 Department of Biotechnology and Genetic Engineering (BG&E), Hazara University Mansehra, Dhodial, Pakistan; 2 Department of Biology, College of Science, Jazan University, Jazan, Saudi Arabia; National Cheng Kung University, TAIWAN

## Abstract

*Chaenomeles japonica* (Thunb.) Lindl., an ornamental species belonging to the family Rosaceae, was molecularly identified from a cultivated population in the Hazara region, Khyber Pakhtunkhwa (KP), Pakistan. Fresh leaf samples were collected from a cultivated garden, and genomic DNA was extracted for molecular analysis. Four chloroplast DNA markers (*matK, rbcLa, trn*A*, and ycf3*) were amplified and sequenced. PCR amplification showed a 100% success rate, generating sequence lengths ranging from 400–1600 bp depending on the marker. BLAST analysis revealed 99–100% sequence identity, 100% query coverage, and E-values of 0.0 when compared with reference sequences available in GenBank, primarily from China, Japan, and the United States. Phylogenetic analysis using Neighbour Joining method demonstrated that the obtained sequences clustered with authenticated *Chaenomeles japonica* accessions, supported by bootstrap values. The results confirm the molecular identity of cultivated *C. japonica* in Pakistan and highlight the utility of chloroplast DNA barcoding markers for accurate species identification of ornamental plants.

## Introduction

*Chaenomeles japonica,* commonly known as Japanese quince (Thunb.) Lindl., is a deciduous shrub belonging to the Rosaceae family [1]. Native to Japan, It is widely cultivated intemperate regions of the world due to its ornamental red to orange flowers, edible fruits, and adaptability to diverse environmental conditions [2]. The fruits, although astringent when raw, are rich in pectin and vitamin C and are commonly processed into jams, jellies, and beverages [3]. Beyond its culinary value, *C. japonica* has been used reported to possess antimicrobial, anti-inflammatory, and antioxidant properties, highligtening its ethnomedicinal importance [4]. These ecological, horticultural, and therapeutic attributed to its global distribution outside its native range [5].

The genus *Chaenomeles* comprises 4–5 species distributed primarily in East Asia, especially China and Japan. Among them, *C. japonica* is one of the most extensively cultivated species because of its hardness and ornamental appeal [2]. It has been introduced into Europe, North America, and other parts of Asia, where it occasionally naturalizes [6,7]. However, species delimitation within the genus remain challenging. Members of Chaenomeles share overlapping morphological traits, including similar leaf shapes, serration patterns, flower coloration, hypanthium structure, and fruit morphology [8]. Furthermore, phenotypic plasticity, environmental influences, horticultural selection, and possible interspecific hybridization complicate accurate identification based solely on morphology. Diagnostic characters may also be absent or incomplete during non-flowering or non-fruiting stages. These limitations highlight the need for integrative approaches combining molecular and morphological data to ensure precise taxonomic resolution [9].

Accurate identification is particularly critical for newly recorded or potentially invasive taxa [10]. Traditional morphological taxonomy can be constrained by environmental variability and developmental stages [11]. In this context, DNA barcoding has emerged as a reliable tool for species identification and phylogenetic inference [12]. Chloroplast markers are commonly used in plant barcoding due to their conserved structure and maternal inheritance. The rbcL gene is characterized by high universality and amplification success, although it typically exhibits relatively low interspecific variability [13]. In contrast, *matK* evolves more rapidly and provides greater discriminatory power at the species level but may show reduced amplification efficiency in some taxa. Non-coding regions such as *trn*A introns often display higher sequence variability, which enhances species resolution, although alignment challenges may arise due to insertions and deletions. Similarly, the chloroplast gene *ycf3* has demonstrated moderate variability and phylogenetic informativeness in angiosperms, though it’s discriminatory capacity may vary among closely related species [14]. The combined use of these loci allows integration of conserved and variable genomic regions, thereby improving identification accuracy and phylogenetic robustness [15].

In Pakistan, the genus *Chaenomeles* has not previously been reported in floristic records, herbarium collections, or Rosaceae checklists. Therefore, the present study integrates morphological assessment with multi-locus DNA barcoding (*matK, rbcL, trnA*, and *ycf3*) to investigate a cultivated specimen suspected to belong to *Chaenomeles.* The specific aim of this study is to provide preliminary molecular identification and phylogenetic placement of the specimen and to confirm the presence of *C. japonica* as a new record for Pakistan. This work contributes to the documentation of non-native ornamental flora and underscores the importance of molecular tools in contemporary plant systematics and biodiversity assessment.

## Materials and methods

### Sample collection

**Fig 1:**
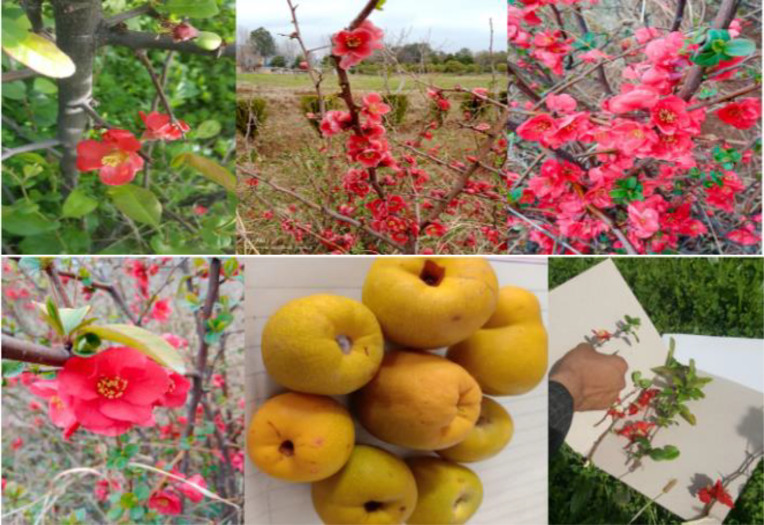
Different morphological major parts of the available *Chaenomeles.*

The plant samples were collected in the form of flowers, fresh leaves and fruits from the cultivated garden of Hazara University (Table 1). The collected samples were properly tagged, pressed, dried, and pasted on the herbarium sheets for future study and record (Fig 1). The fresh leaves were collected in polythene zipper bags; the bags were tagged and stored in a genomic lab at −20^°^C deep freezer for molecular study.

**Table 1:** Collected *Chaenomeles* detail descriptions with coordinates of study area.

Sample	Sampling sites	Latitude °N	Longitude °E
Fresh leaves of query *Chaenomeles*	Hazara University, Mansehra, Khyber Pakhtunkhwa, Pakistan	34.4211°N	73.2502°E

### DNA extraction

For genomic DNA extraction, fresh leaves were ground well with the help of a pestle and mortar in liquid nitrogen. CTAB method with minor changes was used to extracted total gDNA [16]. A pre-warmed CTAB of 800 µl was used for and incubated the ground tissue for 2 hours at 65°C, and samples were vortexed thoroughly [17]. After incubation, phenol chloroform isoamyl alcohol of 600 µl was added, and the homogenate was centrifuged for 20 minutes at 13,000 rpm. Then ice-cold isopropanol of 500 µl was added freeze the sample overnight. Again the samples were centrifuged for 20 minutes at 13,000 rpm. After centrifugation the final products in the form of pellet was washed with 70% ethanol [18]. The extracted genomic DNA was diluted, and checked on gel electrophoresis [19].

### Primer selection and amplification

The most studied and well-known primers shown in Table 2, were selected, and amplified using the prescribed conditions. A 25 µL PCR reaction mixture was prepared in a 200 µL PCR tube, containing ddH_2_O 14 µL, 10 × PCR buffer 2.5 µL, MgCl_2_ 2 µL, dNTPs 2 µL, tDNA 2 µL, Taq Polymerase 0.5 units (Catalogue K0171) and 2 µL of primers (forward and reverse). The PCR steps started with a pre-denaturation for 5 minutes at 94°C, followed by 35 cycles for 30 seconds at 94°C, 40 seconds at 52°C annealing for (*matK*), 56°C for (*rbcla*), 54°C for (*trn*A), and 58°C for (*ycf3*) and about 35 seconds at 72°C for extension. The amplified products were checked on a 1% agarose gel electrophoresis.

**Table 2:** A detailed information of selected markers for the experimental plants identification.

DNA barcodes	Forward and reverse primers	Sources
*mat*K-F *mat*K –R	*5′ATTTTGGAGAAGATTCC3′* *5′CGTGTATATATCTCATTACGC3′*	[20]
*rbc*La-F *rbc*La-R	*5′ATGTCCACAAACAGAGACTAAAGC3′* *5′GTAAAATCAAGTCCACCCACG3′*	[21]
*trn*A-F *trn*A-R	*5′GGTTCAAGTCCCTCTATCCC3′* *5′ATTTGAACTGGTGACACGAG3′*	[22]
*Ycf*3-F *Ycf*3-R	*5′AGAACCGTACTTGAGAGTTTCC3′* *5′CTGTCATTACGTGCGCTATCT3′*	[23]

### Nucleotide sequencing and analysis

The desired amplified regions were sequenced from Macrogn Inc., South Korea through Sanger sequencing. After positive sequencing, good-quality region were used for Basic local alignment (BLASTn) in online ncbi tools [24]. The closely related accessions were downloaded and arranged and analysed through a series of software via BioEdit [25], Multiple Sequence Alignment (MUSCLE) [26], and Clustal-W [27]. The resultant data was processed to compute the Kimura-2-parameter distances for each region by MEGA-11 [28].

### Ethical approval

This study was approved by the Advanced Studies and Research Board and Ethical Review committee of Hazara University, Mansehra, Pakistan (approval granted in the 38^th^ meeting held on October 13, 2021). All procedures were conducted in accordance with institutional guidelines.

## Results

In the current study, four widely used and recommended chloroplast markers were used for initial investigation of experimental plant. The candidate barcode markers showed 100% with respect to amplification, sequencing and similarity with the reference database library sequence of *C. japonica*. The overall performance of the query barcode was higher than 90% in the current study.

### Nucleotide BLAST of candidate barcodes

The query barcode sequences aligned in NCBI BLAST ensure alignment score and homology, and identified the closely related reference database library sequences for subsequent analysis. The nucleotide sequences set consists of sequence length, percent identity, query cover, and E. value of 0.0. After trimming of the targeted sequence along with reference sequences, generating scores in the final dataset as shown in (Table 3).

**Table 3:** A detailed information of indexes obtained through molecular markers analysis.

Query samples	Total seq length	Align seq length	Query cover	E-Value	Identities	Barcodes
*Chaenomeles*	1546	1546	100	0.0	100	*mat*K
*Chaenomeles*	983	983	97	0.0	98	*rbc*La
*Chaenomeles*	806	806	99	0.0	99	*trn*A
*Chaenomeles*	795	795	97	0.0	98	*ycf3*

### Molecular characterizations of DNA barcodes

For molecular characterization, a refined and well-developed FASTA file containing both the query and database accessions were uploaded in MEGA 1.5 software. After analysis, the total aligned sequence length, conserved, variable, parsimony informative and singletons were recorded (Table 4).

**Table 4:** Descriptive information of DNA barcode sequence of both query sequences along with reference database sequences.

Barcodes	Total sites	Conserved	Variable	Parsimony informative	Singleton
*mat*K	953	623	330	329	01
*rbc*La	573	555	18	0	06
*trn*A	460	451	05	02	03
*ycf3*	459	446	05	02	03

### DNA barcode-based similarities indexes

Based on molecular characterisations, the monophyletic clade of query sequences was confirmed with database sequences using MEGA 11 software. The evolutionary history was inferred using different methods like blast similarities and percent identities. The optimal tree is shown in a different clade with bootstrap support. The distances were computed through Kimura 2-parameter method and are in the units of the number of base substitutions per site. All the missing positions were eliminated from the data. In the phylogenetic tree, the query sequence share clade with reference database sequences (Table 5).

**Table 5:** Detailed description of evaluated DNA barcodes in collected *Chaenomeles*, BLAST sequence similarities, identities, Genebank Accessions and geographical locations.

Barcodes	Query samples	Similarities and identity.	Genebank accessions, authorities, and geographical locations
*mat*K	*Chaenomeles-LC919519*	100% *C. japonica*, (Thunb.) Lindl.	LC626016.1, Ogiso, Japan MN506261.1, Sun, China NC035566.1, Jin, USA MZ984211.1, Liu, China
*rbc*La	*Chaenomeles-LC919520*	98% *C. japonica*, (Thunb.) Lindl.	MN506261.1, Sun, China NC035566.1, Jin, USA MZ984211.1, Liu, China MN192833.1, Xu, China
*trn*A	*Chaenomeles-LC919521*	99% *C. japonica*, (Thunb.) Lindl.	MN506261.1, Sun, China NC035566.1, Jin, USA MZ984211.1, Liu, China
*ycf3*	*Chaenomeles-LC919522*	98% *C. japonica*, (Thunb.) Lindl.	MN506261.1, Sun, China NC035566.1, Jin, USA MZ984211.1, Liu, China

### Neighbour-joining tree based on trnA and ycf3

**Fig 2:**
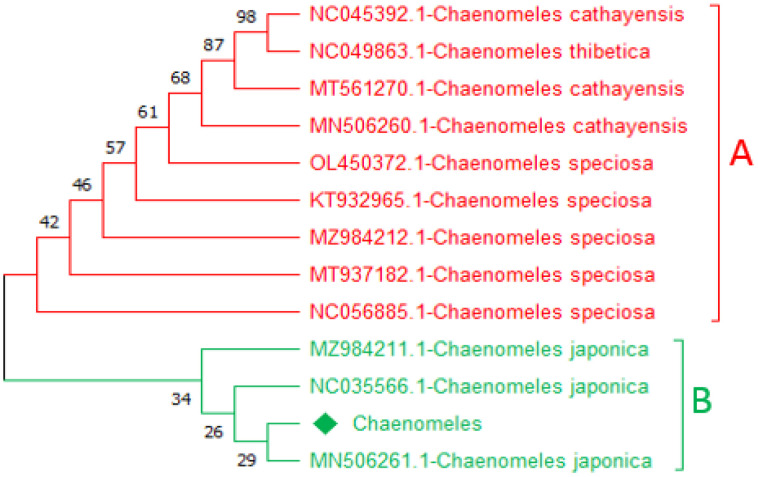
A representative *ycf3-*based tree showing a close relationship of the available sequence of *Chaenomeles*-LC919522 with references *Chaenomeles* accessions MN506261.1-*C. japonica*, NC035566.1-*C. japonica*, and MZ984211.1-*C. japonica.*

**Fig 3:**
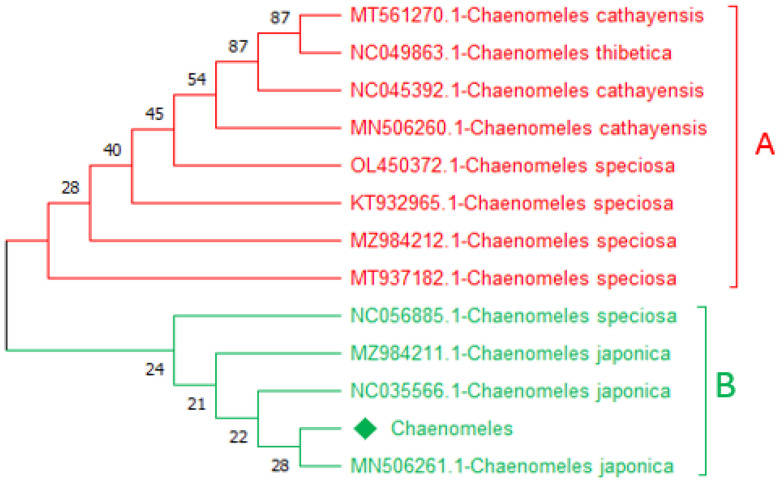
A representative *trn*A-based tree showing the phylogenetic relationship of the available *Chaenomeles-*LC919521 with references *Chaenomeles* accessions MN506261.1-*C. japonica*, NC035566.1-*C. japonica* and MZ984211.1-*C. japonica.*

Construction of a phylogenetic tree based on the NJ method for the query sequence obtained through *trn*A and *ycf3,* and the reference sequences were used to inferred the evolutionary tree. In the phylogenetic tree, two major clades were formed. Based on *ycf3* sequence, the query sequence *Chaenomeles-*LC919522 makes a close relationship with MN506261.1-*C. japonica*, NC035566.1-*C. japonica,* and MZ984211.1-*C. japonica* with bootstrap values of 26, 29, and 34 in clade B. Similarly, based on *trnA* the query sequence *Chaenomeles-*LC919521 makes a close relationship with MN506261.1-*C. japonica*, NC035566.1-*C. japonica*, and MZ984211.1-*C. japonica* with bootstrap values of 21, 22, 24 and 28 in clade B highlighted with green colour. While the remaining members in clade A of both candidate barcodes showed and shared a clade with NC056885.1*-C. speciose*, MT937182.1*-C. speciose*, MZ984212.1-*C. speciose*, KT932965.1*-C. speciose*, OL450372.1*-C. speciose*, MT561270.1*-C. cathayensis*, MN506260.1*-C. cathayensis*, NC045392.1*-C. cathayensis*, and NC049863.1*-C. thibetica* are highlighted with red colour (Figs 2 and 3).

### Neighbour-joining tree based on matK and rbcLa

**Fig 4:**
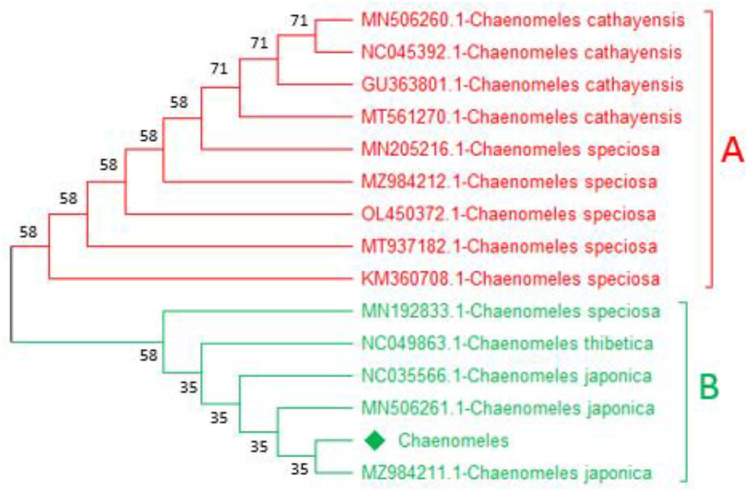
A representative *mat*K-based tree showing the phylogenetic relationship of the available *Chaenomeles*-LC919519 with references *Chaenomeles* accessions MN506261.1-*C. japonica*, NC035566.1-*C. japonica* MZ984211.1-*C. japonica*, and MN192833.1-*C. japonica.*

**Fig 5:**
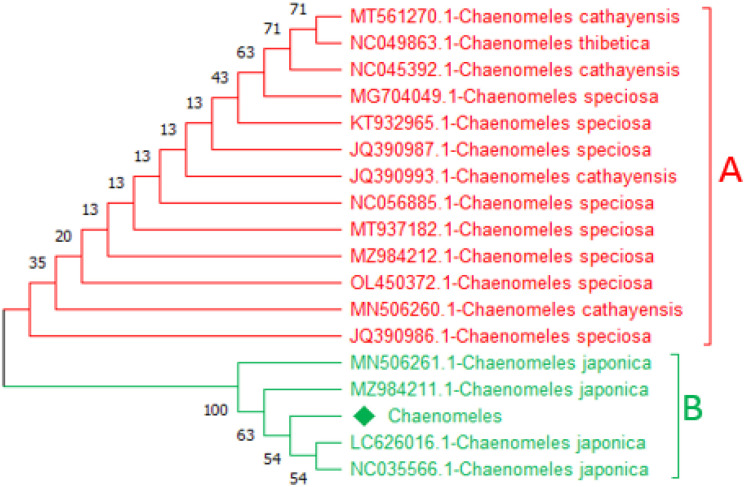
A representative *rbc*La-based tree showing the phylogenetic relationship of the available *Chaenomeles*-LC919520 with references *Chaenomeles* accessions MN506261.1-*C. japonica*, NC035566.1-*C. japonica* MZ984211.1-*C. japonica*, and MN626016.1-*C. japonica* with bootstrap values of 54, 63, and 100.

Similarly, based on the *mat*K and *rbc*La*,* the trees were classified into two clades, i.e., A and B. The query *Chaenomeles,* based on *rbcLa* sequence *Chaenomeles-*LC919520 makes a close relationship with MN506261.1*-C. japonica*, NC035566.1*-C. japonica*, MZ984211.1*-C. japonica*, and MN192833.1-*C. japonica* with bootstrap support of 35 and 58 in clade B highlighted with green (Figs 3 and 4). Similarly, based on the *matK* sequence, the query *Chaenomeles*-LC919519 makes a close relationship with MN506261.1*-C. japonica*, NC035566.1*-C. japonica*, MZ984211.1*-C. japonica*, and MN192833.1*-C.japonica* withbootstrap support of 54, 63, and 100 (Figs 4 and 5). While the rest of the members in clade A of both candidate barcodes showed and shared a clade with NC056885.1*-C. speciose*, MT937182.1*-C. speciose*, MZ984212.1*-C. speciose*, KT932965.1*-C. speciose*, OL450372.1*-C. speciose*, MT561270.1*-C. cathayensis*, MN506260.1*-C. cathayensis*, NC045392.1*-C. cathayensis*, and NC049863.1*-C. thibetica* highlighted with red colour (Figs 4 and 5).

## Discussion

This study provides preliminary molecular evidence supporting the identification of a cultivated specimen as *Chaenomeles japonica* in Pakistan. Rather than constituting floristic confirmation of a wild population, the finding contribute to the molecular characterization of a horticulturally introduced ornamental taxon. The use of four chloroplast loci such as (*mat*K*, rbc*La*, trn*A*,* and *ycf3*) allowed assessment of multilocus barcode performance within the genus *Chaenomeles* and provided comparative insight into their discriminatory capacity.

In family Rosaceae, plastid loci often exhibit limited interspecific divergence, and species delimitation typically benefits from multilocus approaches combining plastid and nuclear markers [29,30]. In the present study, each chloroplast locus contributed partial resolution; however, none independently provided unequivocal species discrimination with strong statistical support.

Among the markers, *mat*K exhibited the highest sequence variability and produced 100% BLAST identity with multiple *C. japonica* reference sequences from Japan, China, and the United States. Its greater number of variable and parsimony-informative sites compared with rbcla is consistent with previous studies identifying matK as one of the more informative plastid barcode in Rosaceae.

In contrast *rbc*La showed lower variability and 98% identity, reflecting its conserved nature. While useful for higher-level taxonomic placement, rbcLa alone provided limited species-level resolution.. The additional loci *trn*A and *ycf3,* demonstrated moderate variability but did not substantially increase phylogenetic support when analyzed independently. Their contribution was complementary rather than decisive. When evaluated collectively, the multilocus dataset increased confidence in sequence similarity results, but phylogenetic support values remained low across several internal nodes [31,32].

Phylogenetic reconstruction using all markers placed the Pakistani specimen within a strongly supported *C. japonica* clade, clustering with reference sequences from China (MN506261.1), Japan (LC626016.1), and the United States (NC035566.1). The clear separation from *C. speciosa, C. cathayensis*, and *C. thibetica* in the sister clade confirms accurate species delimitation. These findings closely match global phylogenetic works on *Chaenomeles,* which consistently recognises *C. japonica* as a distinct lineage characterised by unique chloroplast haplotypes [29,33].

Comparable clustering patterns have been reported from Europe, where barcoding was used to authenticate horticulturally important *Chaenomeles* accessions and distinguish hybrids from pure species [7]. Similarly, North American studies have shown that plastid DNA reliably separates *C. japonica* from naturalised or misidentified specimens in ornamental landscapes [6]. The alignment of the Pakistani specimens with these international lineages confirms that the material introduced in the Hazara region corresponds to genetically validated *C. japonica*.

Morphological identification of *Chaenomeles* species can be challenging due to overlapping vegetative traits and reported -hybridisation among congeners [8,9]. In cultivated settings where reproductive characters may be absent, molecular barcoding provides an objective supplementary tool. In the present study, DNA sequence similarity offered supportive evidence for identification where morphology alone was inconclusive.

However, it is important to emphasize that DNA barcoding functions most effectively when supported by comprehensive reference sampling and multilocus data, ideally including nuclear markers such as ITS or other single-copy nuclear genes.

The confirmation of *C. japonica* as a new record for Pakistan is biogeographically significant. Despite the species widespread cultivation in temperate regions of the world, such as Europe, Central Asia, and North America, its existence in Pakistan has never before been documented in botanical literature, herbarium collections, or natural checklists. Because *C. japonica* is prized for its flowers, fruits, and adaptability, its presence in the Hazara region is consistent with global patterns of horticultural importation [5].

Although *C. japonica* is not widely acknowledge as an aggressive invader, research from Latvia, Romania, and some regions of the United States of America suggest that it can naturalise along riverbanks, forest edges, and semi-natural habitats [6,7]. This highlights the need to monitor its ecological behaviour in Pakistan, especially in sensitive Himalayan foothill ecosystems. Documenting new species is essential for early detection, management planning, and accurate updates to national biodiversity databases [13].

This study illustrates the usefulness of combining DNA barcoding with ongoing horticultural species monitoring and highlights the significance of molecular identification in identifying neglected taxa by confirming *C. japonica* using several independent molecular markers and phylogenetic analysis.

## Conclusion

This study provides preliminary chloroplast DNA evidence supporting the identification–of a cultivated specimen as *C. japonica* within the genus Chaenomeles in Pakistan. The analysis of four plastid loci (*mat*K*, rbc*La*, trn*A*,* and *ycf3*) demonstrated that multilocus chloroplast barcoding can assist in the molecular characterization of ornamental taxa when morphological identification is uncertain or limited.

Among the markers examined, *matK* showed comparatively higher variability, whereas rbcLa displayed lower discriminatory capacity, consistent with its conserved nature. When interpreted collectively, the four loci provided sequence similarity patterns consistent with reference accessions of *C. japonica.* However, phylogenetic reconstruction based solely on plastid data yielded generally low bootstrap support values, limiting confidence in species level resolution.

Because the analysis was conducted on a single cultivated individual and did not incorporate nuclear markers or population-level sampling, the finding should be regarded as tentative molecular evidence rather than definitive taxonomic confirmation. Importantly, this study documents the molecular identity of a cultivated ornamental specimens and does not confirm the occurrence of a naturalized or wild population in Pakistan’s flora.
